# Using maximum weight to redefine body mass index categories in studies of the mortality risks of obesity

**DOI:** 10.1186/1478-7954-12-6

**Published:** 2014-03-17

**Authors:** Andrew Stokes

**Affiliations:** 1 Population Studies Center, University of Pennsylvania, 3718 Locust Walk, McNeil Building, Room 239, Philadelphia, PA 19104, USA

**Keywords:** Body mass index, Maximum weight, Obesity, Mortality, Confounding, Reverse causality, Population attributable fraction

## Abstract

**Background:**

The high prevalence of disease and associated weight loss at older ages limits the validity of prospective cohort studies examining the association between body mass index (BMI) and mortality.

**Methods:**

I examined mortality associated with excess weight using maximum BMI—a measure that is robust to confounding by illness-induced weight loss. Analyses were carried out on US never-smoking adults ages 50-84 using data from the National Health and Nutrition Examination Surveys (1988-1994 and 1999-2004) linked to the National Death Index through 2006. Cox models were used to estimate hazard ratios for mortality according to BMI at time of survey and at maximum.

**Results:**

Using maximum BMI, hazard ratios for overweight (BMI, 25.0-29.9 kg/m^2^), obese class 1 (BMI, 30.0-34.9 kg/m^2^) and obese class 2 (BMI, 35.0 kg/m^2^ and above) relative to normal weight (BMI, 18.5-24.9 kg/m^2^) were 1.28 (95% confidence interval [CI], 0.89-1.84), 1.67 (95% CI, 1.15-2.40), and 2.15 (95% CI, 1.47-3.14), respectively. The corresponding hazard ratios using BMI at time of survey were 0.98 (95% CI, 0.77-1.24), 1.18 (95% CI, 0.91-1.54), and 1.31 (95% CI, 0.95-1.81). The percentage of mortality attributable to overweight and obesity among never-smoking adults ages 50-84 was 33% when assessed using maximum BMI. The comparable figure obtained using BMI at time of survey was substantially smaller at 5%. The discrepancy in estimates is explained by the fact that when using BMI at time of survey, the normal category combines low-risk stable-weight individuals with high-risk individuals that have experienced weight loss. In contrast, only the low-risk stable-weight group is categorized as normal weight using maximum BMI.

**Conclusions:**

Use of maximum BMI reveals that estimates based on BMI at the time of survey may substantially underestimate the mortality burden associated with excess weight in the US.

## Background

Many studies of body mass index (BMI, measured in kg/m^2^) and mortality in older adults find weak or even inverse associations between excess BMI and mortality [1-4]. Several physiologic and behavioral explanations for the paradoxical findings have been proposed [5]. An alternative explanation for the weak or inverse associations identified in prior research is confounding by illness-induced weight loss—also referred to as reverse causality [6-9].

Consistent with the statistical explanation, numerous studies find significantly stronger mortality risks of obesity after implementing measures aimed at reducing reverse causality, such as restricting samples to “healthy” participants and delaying onset of risk for several years after the time of the survey [9,10]. These strategies, however, have been criticized on several grounds: the exclusions lead to eliminating a large proportion of deaths among respondents, thereby reducing the generalizability of findings [11]. Also, pre-existing illness is identified on the basis of respondent self-reports, meaning that individuals with undiagnosed illnesses cannot be excluded. Finally, delaying onset of risk for several years may not be effective at addressing reverse causality, as illness-induced weight loss can begin many years before death [12].

In this study, I investigate the mortality risks of obesity among older adults in the US using an approach that incorporates individual weight histories and is robust to reverse causality. Unlike other methods of addressing reverse causality, the present approach does not require excluding participants or delaying onset of risk. Instead of using BMI at time of survey, I employ a measure of maximum lifetime BMI. The advantage of the latter is that it is not susceptible to fluctuations in BMI related to illness. I also calculate the population attributable fractions for overweight and obesity for US adults implied by the estimated mortality risks.

## Methods

The National Health and Nutrition Examination Surveys (NHANES) provide nationally representative data on health for the US noninstitutional population. I used data from NHANES 3 (1988-1994) and continuous NHANES (1999-2004) to construct the cohort and obtained information on mortality status through the end of 2006 from the National Death Index [13]. The sample was restricted to never-smoking adults ages 50-84. The exclusion of ever-smokers was carried out because smoking is a powerful confounder of the association between BMI and mortality [7,14,15]. After these exclusions and further eliminating individuals with BMI less than 18.5 kg/m^2^ and those with missing data on BMI, education, and mortality status, the final analytic sample consisted of 5,540 individuals. A total of 903 deaths occurred during follow-up in 42,281 person-years.

Demographic variables (gender, race/ethnicity, and educational attainment) and maximum weight were determined by interview. To ascertain maximum weight, NHANES respondents were asked, “Up to the present time, what is the most you have ever weighed?” Respondents were instructed not to include weight during pregnancy. Weight and height at the time of survey were measured by trained personnel in mobile examination clinics and used to calculate BMI at the time of survey. Maximum weight was combined with height measured at the time of survey to calculate maximum BMI. Categories of BMI at time of survey and at maximum were constructed on the basis of the continuous measures. For both variables, I used the standard WHO categories: normal (18.5-25 kg/m^2^), overweight (25.0-30.0 kg/m^2^), obese class 1 (30.0-35.0 kg/m^2^), and obese class 2 (35.0 kg/m^2^ and above). In a small proportion of the data (192 cases), respondents' BMI at time of survey category exceeded their BMI at maximum category and the former was substituted for the latter. Among these individuals, the median difference in the two BMI values was 1.6 units.

Respondents were also categorized into ten different weight trajectories (normal-normal, over-normal, obese 1- normal, obese 2-normal, over-over, obese 1-over, obese 2-over, obese 1-obese 1, obese 2- obese 1, obese 2-obese 2) on the basis of their maximum BMI and BMI at time of survey. For example, an individual who was in the obese class 2 category at their maximum and in the normal weight category at the time of survey would be categorized as “obese 2-normal”.

Mortality rates were calculated as the ratio of the number of deaths to person-years and standardized to the US population in 2000 using five-year age groups between 50-54 and 80-84. Rates were calculated separately based on BMI at maximum and at time of survey as well as for each of 10 weight trajectories defined on the basis of both variables. Cox proportional hazards models with age as the underlying time scale were used to examine the hazard ratios associated with each BMI category relative to the reference category of normal BMI. Hazard ratios were also estimated for each of the 10 weight trajectories using normal BMI at maximum and at time of survey as the reference group. All models were adjusted for gender, race/ethnicity, and educational attainment.

I used the hazard ratios obtained above to estimate population attributable fractions (PAF). These provide an estimate of the percentage of mortality at the population level that is attributable to the combination of overweight and obesity. I use the following formula to estimate PAFs:

(1) $$\mathrm{PA}F_{k}=pd_{k}\left(\frac{HR_{k}-1}{HR_{k}}\right)$$

Equation 1 is the appropriate formula for use with hazard ratios adjusted for confounding [16]. In this equation, PAF_k_ denotes the PAF for the kth level of the risk factor, pd_k_ denotes exposure to risk at level k among deceased individuals, and HR_k_ is the hazard ratio associated with exposure level k. The exposure categories for which PAF_k_ is estimated include overweight, obese class 1, and obese class 2. The total PAF is obtained by summing the PAFs across exposure categories.

All estimates incorporated sampling weights that capture unequal probabilities of selection and nonresponse adjustments and accounted for the complex survey design of NHANES. Analyses were based on anonymous secondary data and therefore did not require approval from an ethics committee. Results were generated using STATA 12 (StataCorp, Texas, USA). Variances were estimated with the SVY routine, which uses Taylor series linearization.

## Results

**Figure 1:**
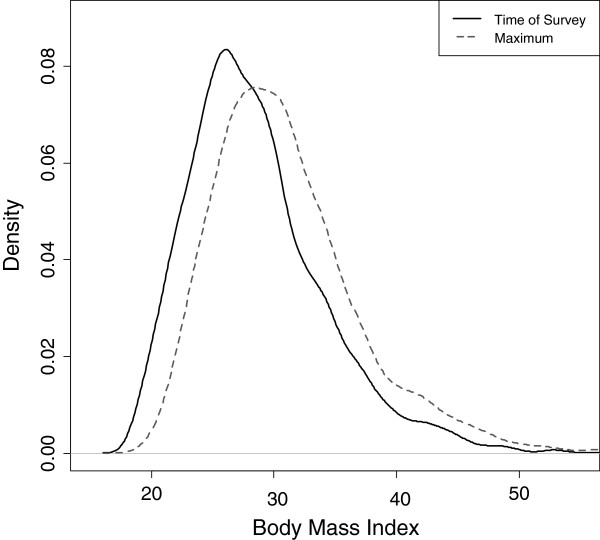
**Distribution of body mass index at time of survey and at maximum among US never-smoking adults ages 50-84.** Distributions are unweighted. Source: National Health and Nutrition Examination Survey.

Figure 1 presents a comparison of the population distributions of BMI measured using time of survey and maximum values. Comparison of the two distributions reveals a greater density at higher BMI values using maximum values (Figure 1).

Descriptive statistics of the study sample, consisting of US adults ages 50-84 who never smoked, are presented in Table 1. Mean age at survey was slightly over 64 years. At the time of the survey, 20% and 12% of adults were in the obese class 1 and obese class 2 categories, respectively. When obesity status was assessed using maximum BMI, the percent obese class 1 and obese class 2 climbed to 27% and 19%.

**Table 1:** Characteristics of US never-smoking adults ages 50-84

	**No.**	**% or mean**
Age at survey, years		64.09
Education		
Less than high school	2,461	28.35
High school or equiv.	1,395	29.02
More than high school	1,684	42.63
Race/ethnicity		
Hispanic	1,371	8.54
Non-Hispanic white	2,944	77.81
Non-Hispanic black	1,079	9.25
Non-Hispanic other	146	4.41
Obesity status at survey		
Normal	1,542	29.70
Overweight	2,171	38.13
Obese class I	1,152	20.00
Obese class II	675	12.18
Obesity status at maximum		
Normal	768	17.09
Overweight	1,991	36.36
Obese class I	1,649	27.44
Obese class II	1,132	19.11
Obesity status: maximum-survey		
Normal - normal	768	17.09
Over - normal	633	10.44
Obese 1 - normal	116	1.80
Obese 2 - normal	25	0.36
Over - over	1,358	25.92
Obese 1 - over	702	10.62
Obese 2 - over	111	1.59
Obese 1 - obese 1	831	15.02
Obese 2 - obese 1	321	4.98
Obese 2 - obese 2	675	12.18
Deceased	903	11.92
Total	5,540	

Categories of BMI are normal weight (18.5-25.0 kg/m^2^); overweight (25.0-29.9 kg/m^2^); obese class 1 (30.0-34.9 kg/m^2^); and obese class 2 (35.0 kg/m^2^ or greater). Percentages and means are calculated using sample weights. Entry years are 1988-2004 with mortality follow-up through 2006. Source: National Health and Nutrition Examination Survey.

Table 1 also shows the population distribution across 10 categories defined using information on BMI at maximum and at time of survey. The majority of individuals (70%) were at their maximum BMI at the time of survey; 17% of individuals were in the normal BMI category both at time of survey and at their maximum BMI, and 26%, 15%, and 12% were overweight, obese class 1, and obese class 2 at both values. The remaining 30% of the population lost weight between their BMI at maximum and time of survey. The majority of individuals in this subpopulation transited between the overweight and normal (10%) or obese class 1 and overweight categories (11%). A small proportion of the population experienced more significant weight loss, with about 2% of individuals going from obese class 2 to normal or overweight and another 2% going from obese class 1 to the normal category.

Cox proportional hazards models predicting mortality for each of the two categorical measures of BMI are presented in Table 2. The results show a much stronger relationship using maximum values. In the specification using BMI at time of survey, the hazard ratios for obese class 1 and obese class 2 were only moderately associated with mortality and were not significant (obese class 1: 1.18 [95% confidence interval (CI), 0.91-1.54); obese class 2: 1.31 [95% CI, 0.95-1.81]). However, in the model using maximum BMI, both categories of obesity were strongly and significantly related to mortality (obese class 1: 1.67 [95% CI, 1.15-2.40]; obese class 2: 2.15 [95% CI, 1.47-3.14]).

**Table 2:** Hazard ratios for mortality from all causes according to body mass index at time of survey and body mass index at maximum

	**BMI, time of survey**		**BMI, maximum**	
**BMI category (kg/m**^**2**^**)**	**Hazard ratio**	**95% CI**	**Hazard ratio**	**95% CI**
Normal	1.00		1.00	
Overweight	0.98	(0.77-1.24)	1.28	(0.89-1.84)
Obese class 1	1.18	(0.91-1.54)	1.67 **	(1.15-2.40)
Obese class 2	1.31	(0.95-1.81)	2.15 ***	(1.47-3.14)

BMI: body mass index. See Table 1 for definitions of BMI categories. The sample includes never-smoking persons ages 50-84. Entry years are 1988-2004 with mortality follow-up through 2006. Hazard ratios are derived from Cox proportional hazards models that adjust for gender, race/ethnicity (non-Hispanic white, non-Hispanic black, Hispanic, other), and educational attainment (less than high school, high school, some college, or greater). Age at exposure is specified as analysis time. The reference category in both regressions is the normal category. All estimates are weighted and account for complex survey design. Source: National Health and Nutrition Examination Survey.

***p < 0.001; **p < 0.01; *p < 0.05.

Kaplan Meier survival curves by category of BMI also reveal more substantial differences in survival across BMI categories using maximum values. A notable difference between the two sets of results is the improved survival of individuals in the normal BMI category when maximum values are used.

Table 3 again shows the hazard ratios for BMI at maximum and at time of survey (these results appear in the final row and column of the table). However, Table 3 has two additional elements. First, it includes age-standardized mortality rates (expressed as deaths per 1,000 person-years) associated with categories of BMI at maximum and at time of survey. Second, it shows age-standardized mortality rates and hazard ratios for each combination of BMI at maximum and time of survey. This information is arrayed in a matrix with the rows identifying categories of BMI at time of survey and columns identifying BMI at maximum. Cells below the diagonal are empty because BMI at time of survey is always equal to or less than BMI at maximum.

**Table 3:** Age-standardized all-cause mortality rates (per 1,000 person-years) and hazard ratios for mortality from all causes according to combinations of body mass index at time of survey and body mass index at maximum

		**BMI, maximum**													
**BMI, time of survey**		**Normal**			**Overweight**			**Obese class 1**			**Obese class 2**			**Pooled across BMI, maximum**	
**Normal**	MR	7.17	(4.58-9.76)	MR	14.18	(8.00-20.37)	MR	16.53	(10.21-22.85)	MR	66.56	(17.41-115.70)	MR	10.41	(7.92-12.91)
	HR	1.00		HR	1.69	(1.12-2.56)	HR	2.69	(1.67-4.33)	HR	4.97	(2.01-12.27)	HR	1.00	
**Overweight**				MR	7.84	(6.06-9.61)	MR	15.22	(10.81-19.64)	MR	22.23	(12.48-31.99)	MR	10.37	(8.48-12.25)
				HR	1.10	(0.76-1.60)	HR	1.76	(1.16-2.66)	HR	3.06	(1.72-5.44)	HR	0.98	(0.77-1.24)
**Obese class 1**							MR	12.55	(8.12-16.98)	MR	17.59	(12.19-22.99)	MR	13.83	(10.00-17.65)
							HR	1.48	(0.98-2.24)	HR	2.28	(1.54-3.36)	HR	1.18	(0.91-1.54)
**Obese class 2**										MR	14.70	(11.00-18.40)	MR	14.70	(11.00-18.40)
										HR	1.85	(1.18-2.89)	HR	1.31	(0.95-1.81)
**Pooled across**	MR	7.17	(4.58-9.76)	MR	9.61	(7.51-11.70)	MR	13.87	(10.64-17.10)	MR	16.92	(13.70-20.13)			
**BMI, time of survey**	HR	1.00		HR	1.28	(0.89-1.84)	HR	1.67	(1.15-2.40)	HR	2.15	(1.47-3.14)			

BMI: body mass index; MR: mortality rate; HR: hazard ratio. See Table 1 for definitions of BMI categories. The sample includes never-smoking persons ages 50-84. Entry years are 1988-2004 with mortality follow-up through 2006. Mortality rates are age-standardized to the US 2000 Census using five-year age-groups between 50-54 and 80-84. The final row and column correspond to mortality rates pooled across BMI at the time of survey and across maximum BMI categories, respectively. Hazard ratios are derived from separate calculations in which adjustment is made for gender, race/ethnicity (non-Hispanic white, non-Hispanic black, Hispanic, other), and educational attainment (less than high school, high school, some college, or greater) using Cox proportional hazards models. Age at exposure is specified as analysis time in all models. All estimates are weighted and account for complex survey design. Source: National Health and Nutrition Examination Survey.

The lowest mortality rates are generally along the diagonal of the matrix corresponding to persons with stable or increasing weight. Those with the lowest mortality rates were individuals of normal weight at their maximum and survey values (7.17 [95% CI, 4.58-9.76]) (measured by deaths per 1,000 person-years), followed by individuals who were overweight (7.84 [95% CI, 6.06-9.61]) or obese class 1 (12.55 [95% CI, 8.12-16.98]) at both their maximum and survey values. Mortality rates were consistently higher in subgroups above the diagonal of the matrix—individuals who lost weight between their BMI at maximum and time of survey. The population subgroups with the highest mortality rates were those that exhibited the most weight loss, including those that went from obese class 2 to normal and overweight and individuals that went from obese class 1 to normal weight. Although the mortality rates were very large in the groups that lost the most weight, the proportion of the population in these groups was small. Only about 2% of individuals transited from obese class 2 to normal or overweight between measurements (Table 1).

**Figure 2:**
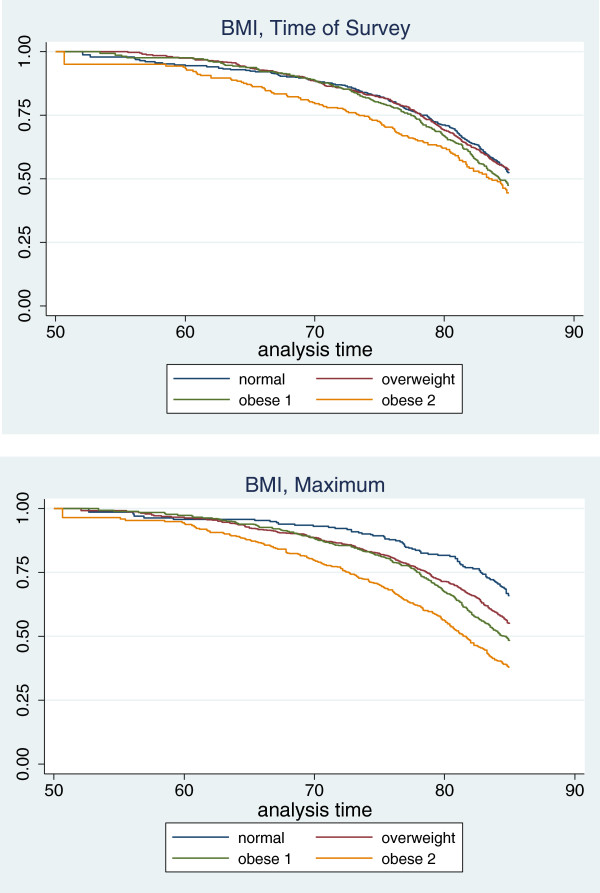
**Kaplan Meier curves for categories of BMI at time of survey and at maximum.** Categories of BMI are normal weight (18.5-25.0 kg/m^2^); overweight (25.0-29.9 kg/m^2^); obese class 1 (30.0-34.9 kg/m^2^); and obese class 2 (35.0 kg/m^2^ or greater). The sample includes persons ages 50-84 who never smoked. Entry years are 1988-2004 with mortality follow-up through 2006. Estimates are weighted and account for complex survey design. Source: National Health and Nutrition Examination Survey.

Table 3 also shows that the mortality rate for normal weight individuals was higher when the category is constructed using BMI at time of survey compared to BMI at maximum (10.41 [95% CI, 7.92-12.91] versus 7.17 [95% CI, 4.58-9.76]). This is consistent with findings from Figure 2 of improved survival among those in the normal category when using BMI at maximum versus BMI at time of survey.

Examination of mortality rates for combinations of BMI at maximum and time of survey reveals the source of the discrepancy. Using BMI at maximum, the normal category only includes stable normal-weight individuals. The mortality rate in this group (7.17) was lower than for any other group in Table 3. In contrast, the normal category defined using BMI at time of survey combines the low-risk stable-weight individuals with high-risk individuals that have experienced weight loss. Thirteen percent of individuals classified as normal using time of survey values were at one point in their lives either overweight or obese (Table 1). Mortality rates among groups that lost weight were substantially greater: 14.18, 16.53, and 66.56 for individuals that were overweight, obese 1, and obese 2 in their past and normal weight at time of survey. The contamination of the normal weight category when it is defined using BMI at time of survey explains why the mortality risks of overweight and obesity grew stronger after substituting maximum BMI for BMI at time of survey in Table 2.

Table 4 shows population attributable fractions for overweight and obesity based on BMI at survey and at maximum. Category-specific and overall PAFs are given. Using BMI at survey, an estimated 5.42% of deaths were attributable to the combination of overweight and obesity, whereas using maximum BMI, the attributable risk was substantially greater, at 32.58%.

**Table 4:** Population attributable fractions estimated using body mass index at time of survey and body mass index at maximum

	**BMI, time of survey**			**BMI, maximum**		
**BMI category (kg/m**^**2**^**)**	**Pd (%)**	**HR**	**PAF (%)**	**Pd (%)**	**HR**	**PAF (%)**
Normal	29.77	1.00	0	11.76	1.00	0
Overweight	36.64	0.98	-0.75	32.92	1.28	7.20
Obese class 1	21.11	1.18	3.22	31.50	1.67	12.64
Obese class 2	12.47	1.31	2.95	23.82	2.15	12.74
Total			5.42			32.58

BMI: body mass index; Pd: proportion exposed among decedents (%); HR: hazard ratio; PAF: population attributable fraction. See Table 1 for definitions of BMI categories. The PAF for each exposure category is calculated using Equation 1 in the text. PAFs are summed across exposure categories to obtain the overall PAF. Calculations are based on the sample of never-smoking adults ages 50-84.

## Discussion

Among older never-smoking adults in the US, use of maximum values for assessing the mortality risks of overweight and obesity yields much stronger associations between excess weight and mortality than using BMI at the time of survey. The analysis of the percentage of mortality attributable to overweight and obesity indicates that use of BMI at the time of survey may significantly underestimate the associated burden of excess weight in the US. Attributable mortality is substantially higher in the analysis using maximum values—33% compared to 5%.

The discrepancy in results relates to who is classified as normal weight across the two measures. This is clearly revealed in examining mortality rates for combinations of BMI at maximum and time of survey. When BMI is assessed at time of survey, the normal weight category includes those who have lost weight from their maximum BMI and are at significantly higher risk for death. Assessment of BMI using maximum values removes this source of confounding, as the reference group is restricted to individuals whose BMIs never exceeded the normal weight category. In addition, individuals who were formerly overweight or obese are included in these categories even if they have lost weight subsequently. This further increases the risks of overweight and obesity relative to the normal category.

Mortality risks were higher in the present study among those subpopulations that lost weight between their maximum and baseline values. This finding is consistent with prior studies that have also identified weight loss as a strong risk factor for mortality [8,17-20]. One explanation for this finding is that most weight loss is associated with illness, masking any beneficial effects of lifestyle modification. A British study that investigated weight loss and mortality found that among individuals losing weight, 78% lost weight because of ill-health—either unintentionally or intentionally—versus the remaining 22% who lost weight for other reasons [21].

Several prior studies have introduced alternative measures of BMI into analyses of obesity and mortality with the aim of reducing bias due to the effects of reverse causality [10,22,23]. In each case, stronger associations were identified, consistent with the findings from the present study.

Although the present study overcomes a serious issue in the literature on obesity and mortality—confounding by illness—it also has several limitations. First, as maximum weight was self-reported, it may be subject to recall bias. If respondents tend to underreport their maximum weight, some individuals may be incorrectly assigned to a lower BMI category. The effects of this bias on the estimated mortality risks of obesity are unclear, as it may lead to mortality rates being overestimated in both the normal and obese categories. Because the analyses use a categorical measure of BMI, potential for misclassification was reduced. Furthermore, validation studies of weight recall support their validity for use in epidemiological studies [24,25]. A second limitation arises from using height at survey to calculate maximum BMI. Because of the tendency for height loss at older ages, maximum BMI may have been overestimated in some respondents. This would be expected to dilute mortality rates in the overweight and obese categories, leading to more conservative estimates of the mortality risks of obesity. A third source of bias is differential mortality of obese individuals. Some individuals who were heavy in their past may not have survived to the time of the survey to report their maximum weight. This bias also leads to more conservative estimates. In order to address these limitations, future research should replicate the analyses presented here using prospective cohort data containing contemporaneous measures of height and weight across the lifecycle.

Prior assessments of associations between excess weight and mortality underestimate mortality risks because of reverse causality owing to the high prevalence of disease in aging populations. The present study suggests that the impact of overweight and obesity on mortality at the population level is likely much larger than is appreciated. As maximum lifetime BMI is highly predictive of mortality, an additional implication of this study is that individual obesity histories should be ascertained in clinical settings to obtain a more complete understanding of individuals’ mortality risks.

## Competing interests

The author declares no competing interests.
